# Optimal beam arrangement for pulmonary ventilation image-guided intensity-modulated radiotherapy for lung cancer

**DOI:** 10.1186/1748-717X-9-184

**Published:** 2014-08-16

**Authors:** Ruihao Wang, Shuxu Zhang, Hui Yu, Shengqu Lin, Guoqian Zhang, Rijie Tang, Bin Qi

**Affiliations:** Departments of Radiotherapy, Cancer Hospital of Guangzhou Medical University, 78 Hengzhigang Road, Guangzhou, 510095 China; Departments of Radiology, Cancer Hospital of Guangzhou Medical University, 78 Hengzhigang Road, Guangzhou, 510095 China; Departments of Radiation Oncology, Cancer Hospital of Guangzhou Medical University, 78 Hengzhigang Road, Guangzhou, 510095 China

**Keywords:** Intensity-modulated radiotherapy, Pulmonary ventilation, Four-dimensional computed tomography, Plan optimization

## Abstract

**Background:**

The principal aim of this study was to evaluate the feasibility of incorporating four-dimensional (4D)-computed tomography (CT)-based functional information into treatment planning and to evaluate the potential benefits of individualized beam setups to better protect lung functionality in patients with non-small cell lung cancer (NSCLC).

**Methods:**

Peak-exhale and peak-inhale CT scans were carried out in 16 patients with NSCLC treated with intensity-modulated radiotherapy (IMRT). 4D-CT-based ventilation information was generated from the two sets of CT images using deformable image registration. Four kinds of IMRT plans were generated for each patient: two anatomic plans without incorporation of ventilation information, and two functional plans with ventilation information, using either five equally spaced beams (FESB) or five manually optimized beams (FMOB). The dosimetric parameters of the plans were compared in terms of target and normal tissue structures, with special focus on dose delivered to total lung and functional lung.

**Results:**

In both the anatomic and functional plans, the percentages of both the functional and total lung regions irradiated at V_5_, V_10_, and V_20_ (percentage volume irradiated to >5, >10 and >20 Gy, respectively) were significantly lower for FMOB compared with FESB (P < 0.05), but there was no significant difference for V_30_ (P > 0.05). Compared with FESB, a greater degree of sparing of the functional lung was achieved in functional IMRT plans with optimal beam arrangement, without compromising target volume coverage or the irradiated volume of organs at risk, such as the spinal cord, esophagus, and heart.

**Conclusions:**

Pulmonary ventilation image-guided IMRT planning with further optimization of beam arrangements improves the preservation of functional lung in patients with NSCLC.

## Background

Radiation therapy (RT) plays a significant role in the curative treatment of surgically inoperable non-small cell lung cancer (NSCLC). Radiation pneumonitis (RP) is the most common complication of RT for NSCLC, and its occurrence and severity are closely correlated with the mean lung dose, total lung volume irradiated to >20 Gy (V_20_), location of the tumor, pulmonary function, and simultaneous or sequential chemotherapy [1–4]. Graham et al. [5] showed that when the V_20_ was <22%, the incidence of RP within 2 years was zero; however, the incidences of RP within 2 years increased to 7%, 13%, and 36% when the V_20_ percentages were 22–31%, 32–40%, and >40%, respectively. This risk of RP could be reduced by up to 10% by using intensity-modulated radiotherapy (IMRT), without compromising tumor-dose delivery [6]. Nevertheless, ways of minimizing RT-induced side effects such as RP, while continuing to achieve reasonable local control of NSCLC, remain a challenge for radiation oncologists.

Information on pulmonary function provided by perfusion and/or ventilation imaging has been shown to be important in evaluating pulmonary toxicity after RT for NSCLC [7–10]. Several techniques exist for pulmonary ventilation imaging, including single photon emission computed tomography and X-ray CT (SPECT-CT) [7, 8], hyperpolarized helium-3 magnetic resonance imaging (^3^He-MRI) [9, 10], and inert xenon CT (Xe-CT) [11]. However, although all these imaging modalities can provide useful ventilation information, each has serious drawbacks, such as high cost, low resolution, long scan time and/or low accessibility [12].

Four-dimensional (4D) CT imaging is an exciting new form of ventilation imaging, which consists of three-dimensional (3D) CT images resolved into different phases of the breathing cycle [13]. 4D-CT is becoming widely available and has shown great promise for treatment planning. Because 4D-CT data are relatively easy to acquire during treatment planning, calculating ventilation maps from 4D-CT data only involves additional image processing, and does not add any extra dosimetric or monetary cost to the patient. Moreover, 4D-CT ventilation imaging has higher resolution, a shorter scan time and is more accessible than other existing techniques. Previous studies have investigated the incorporation of 4D-CT ventilation imaging into RT treatment planning [8, 14–16]. For example, Castillo et al. [8] examined different ways of calculating ventilation from 4D-CT data to estimate local volume changes, and compared the results with those obtained clinically from SPECT ventilation. Ding et al. [14] explored the changes in lung ventilation after RT, and Yamamoto et al. [15] and Yaremko et al. [16] discussed the idea of designing treatment plans to avoid high-ventilation areas of the lung. However, most of these studies considered treatment planning based on fixed-beam arrangements, and to the best of our knowledge, no studies have compared the use of different beam arrangements.

The primary purpose of this study was to investigate the feasibility of combining IMRT with ventilation maps calculated from 4D-CT data, and to compare the benefits of optimal and fixed-beam arrangements in IMRT for NSCLC.

## Methods

### Patient selection

This study was approved by the Institutional Review Board (IRB) at the Affiliated Tumor Hospital of Guangzhou Medical University. Informed consent was obtained from each patient, in accordance with the IRB regulations.

Sixteen consecutive patients with Stage III-IV and recurrent NSCLC who previously underwent IMRT were included. The characteristics of the patients are presented in Table 1. The 4D-CT data sets were obtained using a 16-detector row spiral CT scanner (Lightspeed 16; GE Medical Systems, Milwaukee, WI, USA) in cine mode with a 2.5-mm slice covering the entire chest. A medical physicist was present during each CT scan to ensure that the patient’s breathing was not erratic. To compute the pulmonary ventilation, the paired 4D-CT images at the peak-exhale and peak-inhale phases were selected in this study. Additional details regarding the use of 4D-CT have been published in our previous work [17].

**Table 1:** **Patient characteristics**

Characteristic	n (%)
Age (yr)	
median	55
range	38–73
Sex	
male	14 (87.5)
female	2 (12.5)
Histologic type	
NSCLC, adenocarcinoma	5 (31.2)
NSCLC, squamous cell carcinoma	11(68.8)
Stage	
IIIA	3 (18.8)
IIIB	4 (25.0)
IV	7 (43.7)
Recurrent	2 (12.5)
Location	
RUL	4 (25.0)
RLL	5 (31.2)
LUL	4 (25.0)
LLL	3 (18.8)
PTV (cm^3^)	
median	596
range	176-3647

*Abbreviations*: *NSCLC* Non-small cell lung cancer, *RUL* Right upper lobe, *RLL* Right lower lobe, *LUL* Left upper lobe, *LLL* Left lower lobe, *PTV* Planning target volume.

### 4D-CT pulmonary ventilation imaging

**Figure 1:**
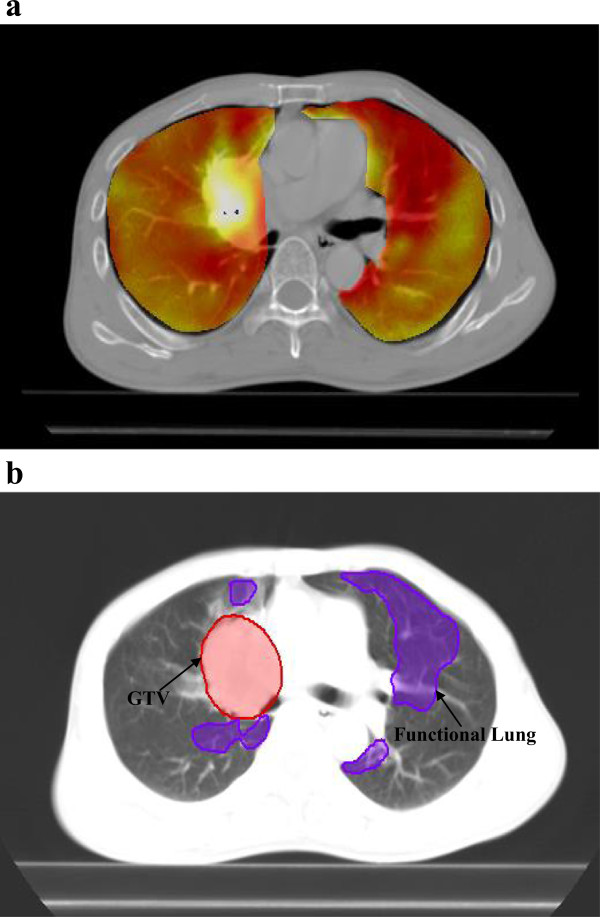
**The functional lung of ventilation maps in a case. (a)** Fusion imaging of CT image and pulmonary ventilation extracted from the generated deformation field; and **(b)** highly functional lung regions contoured on **(a)**. In this study, regions in the top 30% for ventilation in the ventilation image were defined as functional lung structures to avoid in the functional IMRT plans.

Processing 4D-CT pulmonary ventilation imaging involved three steps. Firstly, paired 4D-CT images obtained at the peak-exhale and peak-inhale phases were transferred to our self-developed ventilation imaging software (ZHANGShuxu 4D-CT LF, V1.0) for ventilation computation. Deformable image registration (DIR) was then used to link corresponding lung volume elements between the peak-exhale and peak-inhale 4D-CT data sets, to produce a displacement vector field (DVF). We used 3D-image registration based on a B-spline elastic algorithm, the geometric accuracy of which has been validated previously [18]. 3D-image registration was used to create a voxel-by-voxel displacement map showing the motion of the lung tissue as a function of respiratory state. Finally, a 4D-CT ventilation image was created by quantitative analysis of the DVF. In this study, the Jacobian determinant of deformation [19] was used to measure the degree of regional lung expansion that was directly related to specific volume change. The Jacobian determinant of DVF was calculated for each voxel in the lung and used to represent local tissue expansion (or contraction). The regional ventilation was given by:
1

where the function represents the displacement vector, mapping the voxel at each point of a peak-exhale image to the corresponding position on a peak-inhale image. In addition, the functional lung of ventilation mapping procedure was shown in Figure 1.

### IMRT planning

The Oncentra treatment planning system, version 4.1 (Nucletron V.B., Veenendaal, The Netherlands) was used to delineate the target volumes and organs at risk (OARs) and for IMRT planning and dose calculation. Both target volumes and OARs, including the spinal cord, esophagus, heart, and lungs, were contoured and approved on peak-exhale 4D-CT images by an attending physician. The gross tumor volume to clinical target volume (CTV), and CTV to planning target volume (PTV) margins were 6–8 mm and 8 mm, respectively. Planning OAR volumes were generated for the spinal cord and esophagus by adding 5.0-mm isotropic margins. In addition, functional lung regions were defined from 4D-CT ventilation images for functional planning, as described for other IMRT studies for NSCLC [16]. In this study, regions in the top 30% for ventilation in the 4D-CT-based ventilation image were set as functional lung structures to avoid in the functional IMRT plans. Moreover, the total lung and functional lung represented the total volume and the functional part of the sum of both lungs.

All the IMRT plans were designed to deliver 66 Gy in 33 fractions to cover 95% of the PTV. The radiation dose distributions were calculated using the collapsed cone algorithm on a graphics processing unit (GPU). Four independent treatment plans were designed for each case in this study. First, two anatomically-constrained plans was generated without the incorporation of pulmonary ventilation information, with the main goal of minimizing the volumes of total normal lung and other normal tissues that were irradiated above their tolerance levels (Table 2). Second, two functionally-constrained plans incorporating pulmonary ventilation information was generated with additional constraints to reduce the V_10_ and V_20_ of the identified functional lung regions (Table 2). In addition, dosimetric differences between beam arrangements were evaluated using five equally spaced beams (FESB) positioned every 72° from the anterior and lateral directions, and five manually optimized beams (FMOB) for both the anatomic and functional plans. In plan with FMOB, gantry angles were changed to avoid critical OARs, highly functional lung regions and the non-involved total lung regions.

**Table 2:** **IMRT planning goals and constraints**

Structure	Constraint
PTV	Min. dose ≥65 Gy
	Max. dose ≤70 Gy
	Volume receiving ≥66 Gy more than 95%
Total lung	D_mean_ 20 Gy
	Volume receiving ≥20 Gy less than 30%
	Volume receiving ≥30 Gy less than 20%
Spinal cord	Max. point dose ≤40 Gy
Heart	Volume receiving ≥40 Gy less than 100%
	Volume receiving ≥45 Gy less than 67%
	Volume receiving ≥60 Gy less than 33%
Esophagus	Volume receiving ≥55 Gy less than 35%
Skin	Max. dose ≤70 Gy
Functional lung^a^	Volume receiving ≥20 Gy less than 20%
	Volume receiving ≥10 Gy less than 35%

*Abbreviations*: *IMRT* Intensity-modulated radiotherapy, *PTV* Planning target volume, *Min.* Minimum, *Max.* Maximum, *D*_*mean*_ Mean dose. ^a^for functional-constrained plan.

### Data analysis and statistical methods

The quality of the IMRT plans was evaluated by comparing the metrics of PTVs using common dose-volume parameters such as the conformity index (CI), heterogeneity index (HI), mean dose (D_mean_), and total number of monitor units (MUs). Dosimetric parameters including the percentage of lung volume receiving >5 Gy, >10 Gy, >20 Gy, and >30 Gy were calculated for the functional lung (FLV_5_, FLV_10_, FLV_20_, and FLV_30_, respectively) and total lung (TLV_5_, TLV_10_, TLV_20_, and TLV_30_, respectively). Regarding the other critical structures, the maximum dose (D_max_) delivered to the spinal cord, the D_mean_ and D_max_ delivered to esophagus, and the V_40_, V_45_, and V_60_ delivered to the heart were also quantified and compared between the anatomic and functional plans with different beam arrangements.

Statistical analysis was performed using SPSS 19.0 statistical software (SPSS Inc., Chicago, IL, USA). Volumetric and dosimetric parameters for the target volumes and OARs were characterized using descriptive statistics. The dosimetric differences between the application of FESB and FMOB were evaluated for each plan and compared using paired, two tailed *t*-tests. A P value < 0.05 was considered statistically significant.

## Results

All the anatomically-constrained and functional-constrained plans developed in this study were clinically acceptable. There were no significant differences in PTV dosimetric parameters such as CI, HI, and D_mean_ (P > 0.05) between the anatomic and functional plans using FMOB or FESB, as shown in Table 3. In addition, the functional plans resulted in slightly higher MUs than the anatomic plans, as expected given the additional constraints for functional planning, though the differences were not significant.

**Table 3:** **Comparison of PTV parameters between different plans**

	Anatomic plan			Functional plan		
Paremeter	FESB	FMOB	P value	FESB	FMOB	P value
Conformity index^a^	1.01±0.01	1.013±0.02	0.148	1.01±0.02	1.02±0.02	0.617
Homogeneity index^b^	1.06±0.02	1.07±0.03	0.069	1.07±0.02	1.07±0.03	0.837
MUs	485±202	497±207	0.403	492±189	500±223	0.682
Mean dose (Gy)	67.11±0.05	67.18±0.54	0.637	67.09±0.40	67.04±0.42	0.785

*Abbreviations: PTV* Planning target volume, *MUs* Monitor units, *FESB* Five equally-spaced beams, *FMOB* Five manually optimized beams; Data presented as mean±standard deviation.

^a^Conformity index = cover factor × spill factor, where cover factor defined as relative PTV volume receiving ≥66 Gy, and spill factor defined as ratio of PTV receiving ≥66 Gy to total volume receiving 66 Gy.

^b^Homogeneity index = D_5%_/D_95%_ , where D_x%_ is minimum dose in x% of PTV.

Table 4 shows the dosimetric parameters of the total and functional lung for the same beam arrangements between anatomic and functional plan. For FESB, functional plans had lower doses irradiated to the total and functional lung in average compared with those in anatomic plans, with significant reductions (P < 0.05) in lower dose region (V_5_, V_10_, and V_20_); While for FMOB, functional plans resulted in slightly lower doses to the total and functional lung than those in anatomic plans, though the differences were not statistically significant (P > 0.05).

**Table 4:** **Comparison of dosimetric parameters for total and functional lung between anatomic and functional plan for the same beam setups**

Beam setup		Total lung				Functional lung			
	Plan	TLV _5_	TLV _10_	TLV _20_	TLV _30_	FLV _5_	FLV _10_	FLV _20_	FLV _30_
FESB	Anatomic plan	61.7±12.9	46.7±11.4	30.7±5.3	21.2±5.3	54.2±21.7	42.5±22.1	26.3±20.7	17.0±12.6
	Functional plan	60.3±12.8	43.5±11.3	29.1±6.7	20.9±5.6	52.4±20.7	37.1±12.2	21.1±5.8	16.7±6.8
	P value	0.002	0.001	0.051	0.232	0.005	0.029	0.018	0.911
FMOB	Anatomic plan	35.0±12.5	29.1±9.8	23.3±7.1	20.1±6.4	30.2±18.5	24.1±11.5	17.8±7.5	16.2±7.0
	Functional plan	33.3±10.1	27.6±8.5	23.3±6.9	20.1±6.1	29.2±18.3	23.0±14.8	16.7±9.7	14.7±9.5
	P value	0.395	0.226	0.945	0.904	0.757	0.575	0.234	0.258

*Abbreviations: TLVx* Percentage of volume of total lung receiving > x Gy, *FLVx* Percentage of volume of functional lung receiving > x Gy, *FESB* Five equally-spaced beams, *FMOB* Five manually optimized beams, Data presented as mean±standard deviation.

**Figure 2:**
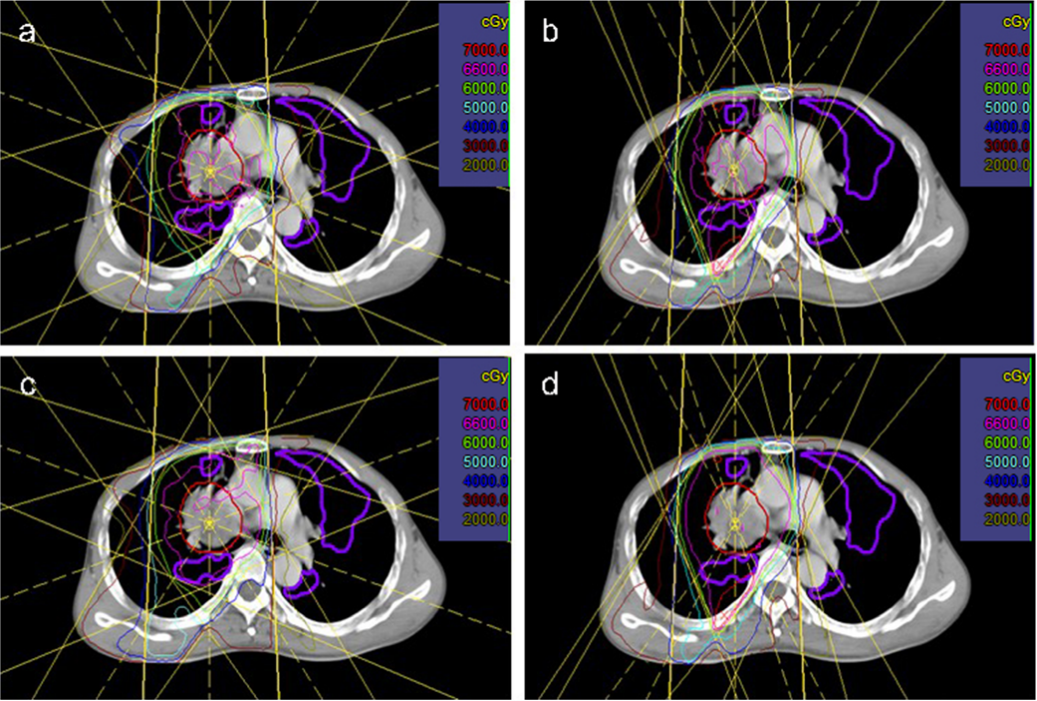
**Comparison of the dose distribution between an anatomic plan with (a) five equally-spaced beam arrangements; and (b) five manually optimized beam arrangements; a functional plan with (c) five equally-spaced beam arrangements; and (d) five manually optimized beam arrangements.**

**Figure 3:**
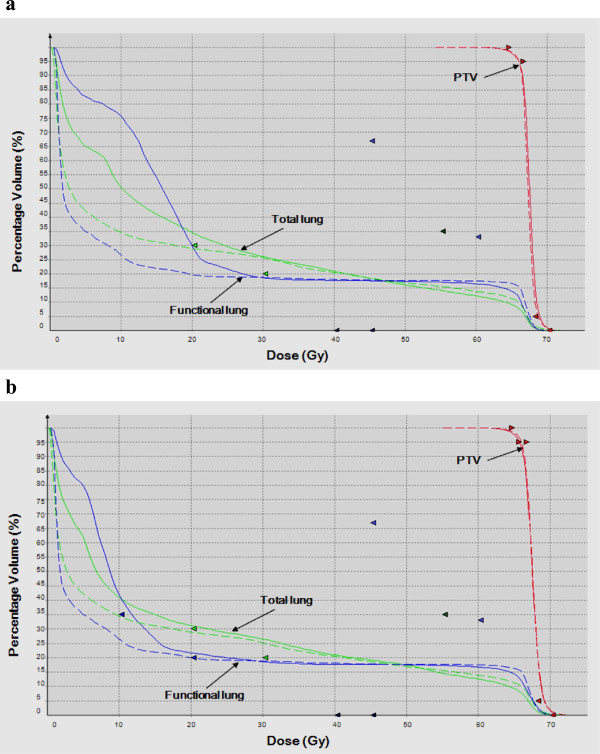
**Dose-volume histogram (DVH) changes in planning target volume (PTV), total lung, and functional lung in a sample case. (a)** DVH of an anatomic-constrained IMRT treatment plan with five equally-spaced beam arrangements (solid lines) and five manual optimization beam arrangements (broken lines). **(b)** DVH changes are also shown in a functional-constrained IMRT plan using highly functional lung as an avoidance structure with different beam arrangements.

Figure 2 shows the isodose distributions for both anatomic and functional plans and for both beam arrangements as an example. Both anatomic and functional plans showed that the highly functional lungs were spared in FMOB, compared with FESB. In particular, the 20- and 30-Gy isodose curves were significantly distorted and moved out of the left lung, which contained large part of highly functional lung. The dosimetric parameters for the total and functional lung regions between different beam arrangements are shown in Tables 5 and 6. In both the anatomic and functional plans, V_5_, V_10_, and V_20_ in the total and functional lung regions were significantly lower in FMOB, compared with FESB (P < 0.05), though there was no significant difference for V_30_ (P > 0.05). The same results were demonstrated using a dose-volume histogram (DVH) (Figure 3).

**Table 5:** **Comparison of dosimetric parameters for total lung between different beam arrangements**

Beam arrangement	Anatomic plan				Functional plan			
	TLV _5_	TLV _10_	TLV _20_	TLV _30_	TLV _5_	TLV _10_	TLV _20_	TLV _30_
FESB	61.7±12.9	46.7±11.4	30.7±5.3	21.2±5.3	60.3±12.8	43.5±11.3	29.1±6.7	20.9±5.6
FMOB	35.0±12.5	29.1±9.8	23.3±7.1	20.1±6.4	33.3±10.1	27.6±8.5	23.3±6.9	20.1±6.1
P value	<0.001	<0.001	<0.001	0.417	<0.001	<0.001	<0.001	0.231

*Abbreviations*: *TLVx* Percentage of volume of total lung receiving > x Gy, *FESB* Five equally-spaced beams, *FMOB* Five manually optimized beams, Data presented as mean±standard deviation.

**Table 6:** **Comparison of dosimetric parameters for functional lung between different beam arrangements**

Beam arrangement	Anatomic plan				Functional plan			
	FLV _5_	FLV _10_	FLV _20_	FLV _30_	FLV _5_	FLV _10_	FLV _20_	FLV _30_
FESB	54.2±21.7	42.5±22.1	26.3±20.7	17.0±12.6	52.4±20.7	37.1±12.2	21.1±5.8	16.7±6.8
FMOB	30.2±18.5	24.1±12.0	17.8±8.9	16.2±7.0	29.2±18.3	23.0±14.8	16.7±9.7	14.7±9.5
P value	<0.001	<0.001	0.049	0.754	<0.001	<0.001	0.001	0.244

*Abbreviations*: *FLVx* Percentage of volume of functional lung receiving > x Gy, *FESB* Five equally-spaced beams, *FMOB* Five manually optimized beams, Data presented as mean±standard deviation.

The doses to the spinal cord, heart, and esophagus using the different beam arrangements are shown in Table 7. The maximum doses delivered to the spinal cord in the anatomic and functional plans were slightly lower for FMOB compared with FESB, but the difference was not significant (P > 0.05). However, the doses delivered to the esophagus were significantly lower for FMOB than for FESB (P < 0.05); the D_mean_ values for the esophagus in the anatomic plan were 22.0 Gy and 16.3 Gy for FESB and FMOB, respectively, while the equivalent D_mean_ values in the functional plan were 22.7 Gy and 16.2 Gy. In the heart, there were significant differences between the FESB and FOMB plans in terms of V_60_ for both the anatomic and functional plans (P < 0.05), but the differences were not significant for V_40_ and V_45_ (P > 0.05).

**Table 7:** **Comparison of dosimetric parameters for OARs between different beam arrangements**

Parameter	Anatomic plan			Functional plan		
	FESB	FMOB	P value	FESB	FMOB	P value
Spinal cord						
D_max_ (Gy)	37.1±7.1	32.7±13.3	0.101	37.1±7.8	33.0±7.0	0.113
Esophagus						
D_mean_ (Gy)	22.0±10.4	16.3±11.6	0.001	22.7±10.3	16.2±11.6	0.001
D_max_ (Gy)	58.1±9.6	53.4±12.0	0.013	57.6±10.4	53.2±12.4	0.020
Heart						
V_40_	17.2±11.1	21.7±16.3	0.144	17.2±11.7	20.9±15.7	0.167
V_45_	13.1±9.1	17.9±13.5	0.057	13.4±10.1	16.3±13.0	0.194
V_60_	5.9±4.6	10.3±9.8	0.032	5.8±5.0	9.7±9.9	0.048

*Abbreviations*: *OARs* Organs at risk, *D*_*max*_ Maximum dose, *D*_*mean*_ Mean dose, *Vx* Percentage of volume receiving > x Gy, *FESB* Five equally-spaced beams, *FMOB* Five manually optimized beams, Data presented as mean±standard deviation.

## Discussion

Current RT planning aimed at limiting lung toxicity assumes a uniform distribution of pulmonary function, and fails to take account of spatial and temporal patterns. Previous studies demonstrated that the incidence and seriousness of RP were positively correlated with total radiation dose to the lungs [20]. Clarification of the relationship between radiation dose and changes in pulmonary function may help to predict and reduce RT-induced pulmonary toxicity. Decreasing the radiation dose to functional lung areas and directing the rays to the parts with perfusion/ventilation defects may help to protect highly functional lung regions and thus reduce the incidence and seriousness of RP [21].

Lung function can be evaluated by functional imaging modalities such as SPECT, PET/CT and ^3^He-MRI. However, these imaging modalities are not yet widely available in China, and their routine application in clinical practice would be associated with a long scan time and high costs to patients. We therefore assessed the value of acquiring functional information using the newly-reported 4D-CT-based ventilation imaging, which has the advantage that ventilation images can be calculated using only an additional processing step (i.e., DIR).

The results of this study showed that 4D-CT-based ventilation information could be used to reduce the radiation dose to the highly functional areas of the lung, assessed by FLV_5–20_, compared with anatomic planning alone, which was consistent with the results reported in previous published papers [15, 16]. Moreover, our findings indicated that both anatomic and functional treatment plans using FMOB could reduce the functional lung regions exposed to lower doses (FLV_5–20_), without compromising target volume coverage, compared with FESB. In addition to the observed improvements in dose-volume parameters of the functional lung, this study also identified dosimetric improvements in the low-dose zones of total lung, especially in terms of improved V_20_ volume. These results were compatible with that of a recent study investigating functional IMRT treatment planning using 4D-CT images [22]. Functional plans using an optimal beam arrangement aimed at preserving the functional lung did not compromise the DVHs of OARs, such as the spinal cord, esophagus, and heart, which may be an additional important clinical factor. These results also demonstrated that this technique could be applied safely to RT treatments for patients with NSCLC, without exceeding the dose-volume tolerances of OARs.

Although significant dosimetric improvements were observed in the lower-dose regions of functional and total lung, the clinical relevance of these improvements in terms of reducing the risk of RT-induced pulmonary toxicity remains unclear. There is currently insufficient outcome data to confirm the correlation between functional lung dose-volume parameters and pulmonary toxicity endpoints, and further studies are needed to determine if dosimetric reductions to functional lung will translate into clinical benefits for NSCLC patients.

Although, several existing studies have validated the 4D-CT-based ventilation imaging modality [23, 24], its regional physiologic accuracy has not been validated in patients. In addition, temporal changes in regional ventilation to a segment of lung previously impaired by compression from a local tumor might occur during the course of RT. A possible explanation of these changes is that the shrinkage of lung tumor volume in response to IMRT treatment may result in increased ventilation as a result of reopening of the airways [25].

Ongoing studies aim to address the above issues by long-term follow-up of a preliminary patient cohort with known functional lung dose-volume parameters. The physiologic accuracy of 4D-CT-based ventilation imaging will be assessed in these patients by quantifying the impact of temporal changes in ventilation during the course of RT and evaluating pulmonary function and morbidity outcomes after radical RT.

## Conclusions

In conclusion, this study demonstrated the feasibility of functional treatment planning using 4D-CT-based pulmonary ventilation information to identify structures to avoid. The results further indicated the dosimetric benefit of optimal beam arrangement compared with fixed-beam arrangement in IMRT treatment planning, in terms of preserving functional lung at lower radiation doses in patients with NSCLC.
